# Modified Reverse Scan Technique for Abutment Teeth

**DOI:** 10.3390/dj14070412

**Published:** 2026-07-06

**Authors:** Pavel Hyspler, Jiri Borovec, Tatjana Dostalova

**Affiliations:** 1 Dental Department, The Military University Hospital Prague, 16902 Prague, Czech Republic; pavel.hyspler@uvn.cz; 2 Department of Stomatology, 3rd Faculty of Medicine, Charles University and University Hospital Kralovske Vinohrady, 10034 Prague, Czech Republic; jiri.borovec@fnkv.cz; 3 Department of Dentistry, 2nd Medical Faculty and Faculty Hospital Motol, Charles University, 15000 Prague, Czech Republic

**Keywords:** CAD/CAM, computer-aided design, merging error, intraoral scanner, laboratory scanner, 3D cast, deformation of 3D cast

## Abstract

This article introduces a modified reverse scan technique for abutment teeth that incorporates laboratory scanners into a fully digital workflow to enhance accuracy and reduces the impact of stitching and merging errors associated with long-span intraoral scans when fabricating extensive tooth-supported prostheses. This cost-effective, time-saving, and versatile procedure uses a 3D-printed interim tooth-supported prosthesis that is sectioned, bonded intraorally, and scanned with a laboratory scanner to improve the virtual definitive cast. The modification is performed using free software.

## 1. Introduction

The concept of the impression-making process in prosthetic dentistry began in the mid-1800s. This technology is very routine but has a number of shortcomings. Many dental impressions contain defects such as cavities, bubbles, and flow defects. Deformation and volume changes during the setting of the impression materials and plaster are also significant. The use of intraoral scanners eliminates most of these shortcomings, but they have limited applicability for large dental prostheses supported by teeth or implants.

Intraoral scanners are based on several principles. The oldest triangulation technology has been in clinical practice since 1981, while parallel confocal microscopy has been used clinically for a shorter time (since 2009) [1]. Other technologies, such as wavefront sampling and interferometry, are not yet widely used. The reduced accuracy of IO scanners in large prosthetic restorations is due to the principle of creating 3D models of the whole jaw regardless of the acquisition technology. Intraoral scanners only create small 3D models due to the size of the scanning head. These are then merged into a larger model by the software. The accuracy of this software process also depends on the size of the overlapping surfaces [2]. It is proven that the deviation increases with the lengthening of the scan path and with the lack of reference surfaces [3].

To address this issue, various methods have been published to minimize or eliminate these errors [4]. These approaches range from using different scanning-facilitating devices [5,6] or intraoral stereophotogrammetry [7,8] to the use of various instruments without merging errors, such as cone beam computed tomography [9,10] extraoral stereophotogrammetric scanners [11,12] and laboratory scanners [13,14]. However, most of these procedures focus on implant-supported prostheses. This article describes a modification of the reverse scan technique (RST) specifically for abutment teeth. The RST method [15] incorporates laboratory scanners into a fully digital workflow, aiming to eliminate merging errors. Its effectiveness for implants has been proven by both laboratory and patient studies [16].

## 2. Technique

**Figure 1:**
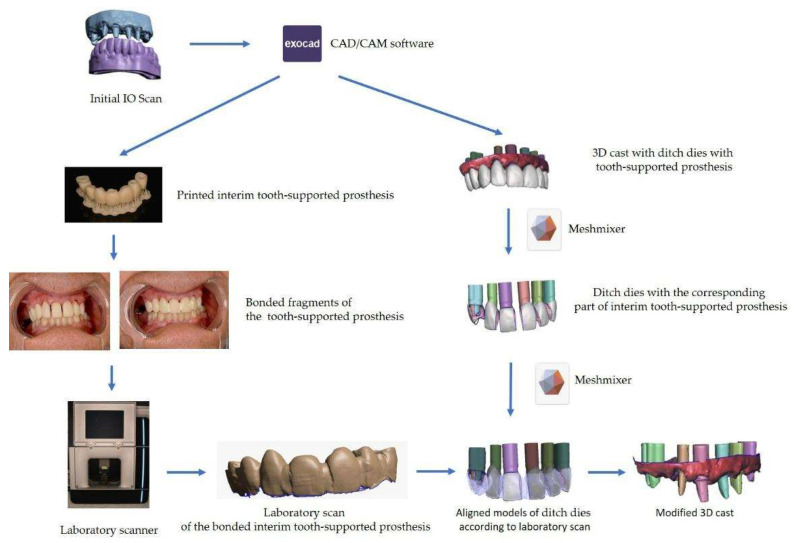
A diagram of the modified reverse scan technique for abutment teeth.

An overview of the method is shown in the diagram in Figure 1. This article uses images from the prosthetic treatment of the following patient: male, age 73, Kennedy classification 1A, treated with a metal–ceramic bridge in the upper jaw in the range of teeth 15–23 with dens pendens 16 and 24, span length of the restoration 52 mm.

**Figure 2:**
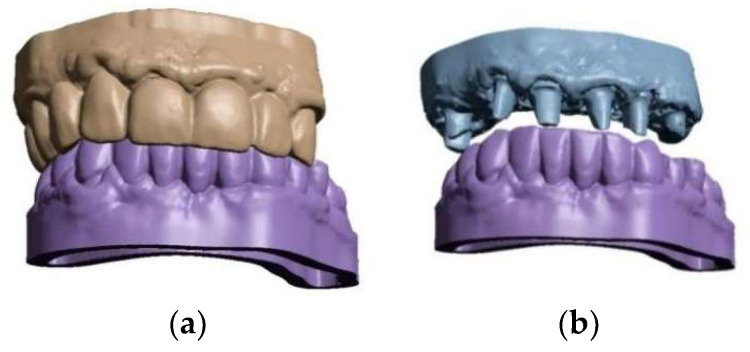
Preliminary scans. (**a**) Pre-preparation; (**b**) preparation.

**Figure 3:**
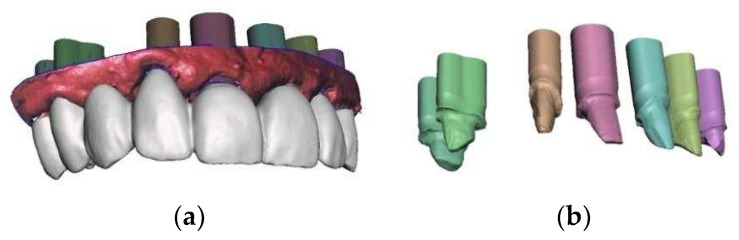
(**a**) 3D digital cast of interim tooth-supported prosthesis with 3D digital master cast with ditch dies; (**b**) ditch dies as separate files.

**Figure 4:**
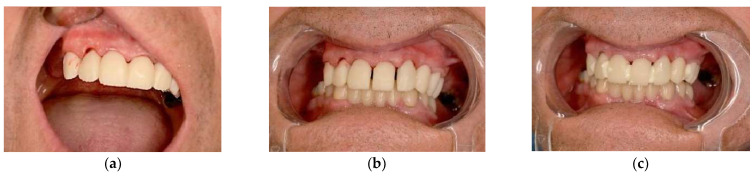
(**a**) 3D-printed interim tooth-supported prosthesis (note gap at tooth 12). (**b**) Sectioned prosthesis. (**c**) Bonded interim tooth-supported prosthesis.

**Figure 5:**
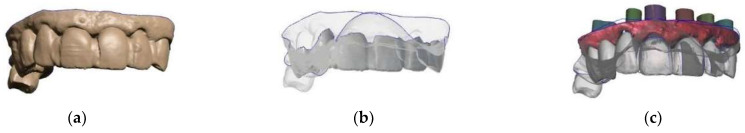
(**a**) Intraoral scan 3D cast of bonded interim tooth-supported prosthesis. (**b**) Merge scan obtained from laboratory scanner combined with intraoral scan of bonded interim tooth-supported prosthesis. (**c**) Merged 3D virtual master cast and interim tooth-supported prosthesis with intraoral scan. Note: Intraoral scan appears transparent in Figure (**b**,**c**).

**Figure 6:**
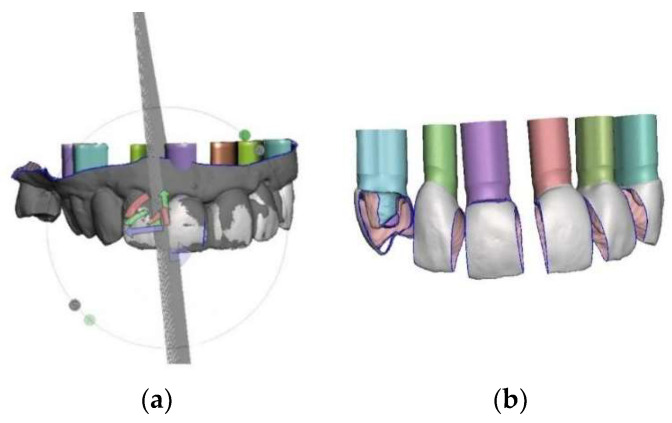
(**a**) Geometric plane of function Plane Cut. (**b**) Sectioned prosthesis combined with ditch dies.

**Figure 7:**
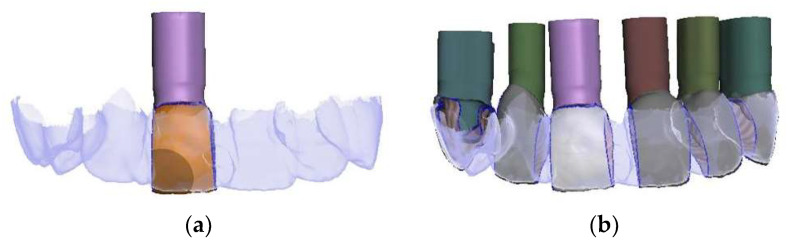
(**a**) Alignment of adequate part of interim tooth-supported prosthesis with ditch die 11 to laboratory scan cast. (**b**) Alignment of all parts. Bonded interim tooth-supported prosthesis obtained by laboratory scanner looks transparent.

**Figure 8:**
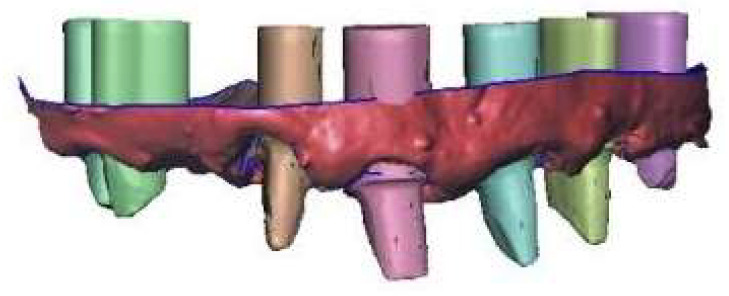
Final modified 3D cast.

1. Scan the pre-preparation and preparation situations with an intraoral scanner (3Shape4, version 22.1.1.; 3Shape A/S, Copenhagen K, Denmark) according to the manufacturer’s recommended procedure without rescanning or image correction (Figure 2).
2. Import the scans into CAD (Computer-Aided Design) software (exocad 3.2; exocad, Darmstadt, Germany) and design the interim tooth-supported prostheses using the following parameters: cement space settings 0.1 mm, marginal offset settings 0.05 mm, and manual margin detection. Export the 3D digital master cast with ditch dies and the 3D cast of the interim tooth-supported prosthesis as separate STL (Standard Tessellation Language) files. Ensure that all 3D casts are in the correct position relative to each other (Figure 3).
3. Produce the interim tooth-supported prosthesis from interim resin (NextDent C&B MFH; 3D Systems Soesterberg, Netherlands) using 3D printing (Original Prusa MedicalOne; Prusa Research, Prague, Czech Republic) (layer thickness 0.025 mm). Carry out cleaning and Post-Curing according to the manufacturer’s recommendations (3 + 2 min 90% ethanol) for 10 min in Medical CW One (Prusa Research, Prague, Czech Republic).
4. Section the interim tooth-supported prosthesis with a cutting disk (405.514.220HP; Rotadent, Vimperk, Czech Republic) into pieces containing only one abutment tooth. Keep the spacing between the sections as minimal as possible to account for the polymerization shrinkage of the splinting material.
5. Fix the fragments to the teeth. If they are not satisfactorily fixed, such as when they fall due to gravity or the gingiva displaces them, use a small amount of temporary cement (RelyX Temp NE, 3M ESPE, Saint Paul, MN, USA).
6. Bond the fragments together with dual-cured composite resin cement (Dentocem; Itena Clinical, Villepinte, France). Light-cure for 20 s. Ensure the pieces are not in direct contact. Touching may cause inaccurate fragment placement. Ensure the adequate adhesion of the splinting material to the interim prosthesis.
7. If occlusal adjustments are needed, trim the interim prosthesis using a dental bur (H79EF.104.040; Komet, Lemgo, Germany) (Figure 4).
8. Scan the patient with the intraoral scanner (3Shape4; 3Shape A/S) with the interim tooth-supported prosthesis in place.
9. Scan the bonded interim tooth-supported prosthesis with the laboratory scanner (AG Map 300; Amann Girrbach AG, Mäder, Austria).
10. Import the STL files of the 3D digital cast, the 3D cast of the interim tooth-supported prosthesis, the 3D cast of the interim tooth-supported prosthesis acquired by the laboratory scanner, and the intraoral scans of the bonded interim tooth-supported prosthesis into the software program Meshmixer 3.5.474 (Autodesk, San Francisco, CA, USA).
11. Align the 3D digital cast of the bonded interim prosthesis obtained with the laboratory scanner to the intraoral scan cast. Set the intraoral scan as the Target.
12. Combine the 3D digital cast of ditch dies, the 3D digital cast of the gingiva, and the 3D digital cast of the interim tooth-supported prosthesis. Select both models in the Object Browser and use the Combine function. Then align the combined cast to the intraoral scan cast (Figure 5).
13. Divide the combined virtual 3D model into fragments containing only one abutment tooth and an appropriate portion of the interim tooth-supported prosthesis (Edit, Plane Cut, Cut Type: Slice [Keep Both], Fill Type: No Fill). Position the cut planes to exclude the bonding material. Adjust the transparency of the intraoral scan cast if needed to facilitate accurate cutting. Separate these into individual 3D casts (Select, Edit…, Separate) (Figure 6).
14. Match these separated 3D digital casts to the 3D cast of the bonded interim tooth-supported prosthesis obtained by the laboratory scanner. Set the scan as the “Target” and align the models using the “Align to Target” function (Solve Iteration 100 and Error tolerance 0). Mark only the outer surface (Figure 7).
15. Combine all ditch dies and the cast of the gingiva using the Combine function and remove all surfaces used for merging. Export the resulting 3D cast as a single STL file (Figure 8).
16. Import the modified 3D cast into the CAD (Computer-Aided Design) program and fabricate the prosthesis using a CAM procedure (computer numerical control milling or 3D printing).

## 3. Discussion

**Figure 9:**
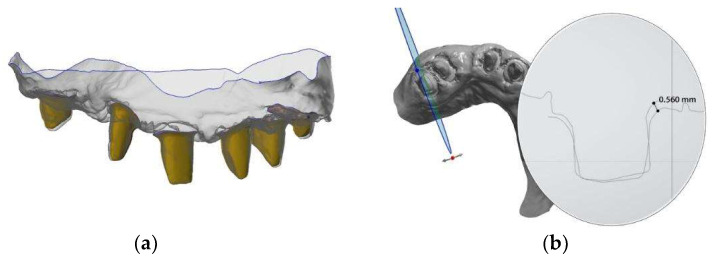
(**a**) A comparison of the preliminary scan with the final modified 3D cast. The preliminary scan is transparent (aligned using Meshmixer). (**b**) A section through the abutment tooth model with the largest error. Note the 0.56 mm change in position (3shape 3D Viewer).

According to our preliminary unpublished in vitro measurements, the presented technique improves the accuracy of abutment tooth positioning by using laboratory scanners and interim tooth-supported prostheses. In the presented patient, the position of tooth 15 had changed by more than 0.5 mm (Figure 9). If the primary scan had been used without modification, the resulting prosthetics would have been clinically unacceptable. The precise measurement of the truth and accuracy of this method is beyond the scope of this tutorial and will be presented in a subsequent article.

The uniqueness of the method lies in its completely innovative use of the outer surface of the bridge to increase accuracy, since laboratory scanners usually have difficulty scanning the inner surfaces of the prosthesis adequately and without unscanned areas. The inner surface is also often contaminated with temporary cement, but the main reason for this is that the surfaces do not match due to the luting spacer and sharp edge relief.

The benefits of a fully digital workflow are well established for both implant- [17] and tooth-supported prosthetics, including increased comfort for both patients and dentists, reduced gag reflex, faster treatment times, and fewer technological steps, which minimizes the potential for human error [18]. These advantages are particularly important when treating pediatric or geriatric patients or patients with disabilities, for whom taking traditional impressions can be very challenging or nearly impossible. Although the need for extensive prosthetic work supported by teeth is less common in modern dentistry, such procedures are still occasionally necessary [19,20].

The procedure saves time and only minimally increases treatment costs, as it employs free software for editing. This seemingly complicated method is simple and surprisingly fast for a well-trained dental technician familiar with using Mashmixer. However, this free software is very popular among dental technicians. It usually takes no more than ten minutes to adjust the scan. This time does not include the time for modeling, manufacturing and bonding the interim tooth-supported prosthesis.

The reverse scan technique for abutment teeth requires further research, particularly the validation of the method and determination of the effects of polymerization shrinkage, thermal shrinkage, digital alignment algorithms, and the effects of 3D printing inaccuracy. It is also important to determine the effect of tooth movement, both physiological and pathological. Empirical experience and measurement results suggest that using the reverse scan technique (RST) is beneficial for both tooth- and implant-supported prostheses when the axial distance between teeth (or implants) exceeds 35 mm in the maxilla and 30 mm in the mandible [15,16], and therefore it seems beneficial to validate all prosthetic work larger than a sextant with this technique. It would be clinically useful to determine the precise distance thresholds for different types of intraoral scanners available on the market.

## 4. Conclusions

This article presents a modified reverse scan technique for abutment teeth that integrates laboratory scanners into a fully digital workflow to improve accuracy and eliminate merging errors when fabricating extensive tooth-supported prostheses.

The method includes preliminary intraoral scans and the design and fabrication of the interim tooth-supported prosthesis using CAD/CAM systems, which is then sectioned, bonded intraorally, and scanned using a laboratory scanner. The final cast is refined using free software. This approach offers significant advantages such as increased precision, reduced clinical errors, and greater patient comfort.

## Data Availability

The data presented in this study are not publicly available due to patient privacy and ethical restrictions. Anonymized data may be made available from the corresponding author upon reasonable request.

## References

[1] van der Meer W.J., Andriessen F.S., Wismeijer D., Ren Y. (2012). Application of Intra-Oral Dental Scanners in the Digital Workflow of Implantology. PLoS ONE.

[2] Logozzo S., Zanetti E.M., Franceschini G., Kilpelä A., Mäkynen A. (2014). Recent Advances in Dental Optics—Part I: 3D Intraoral Scanners for Restorative Dentistry. Opt. Lasers Eng..

[3] Mangano F.G., Hauschild U., Veronesi G., Imburgia M., Mangano C., Admakin O. (2019). Trueness and Precision of 5 Intraoral Scanners in the Impressions of Single and Multiple Implants: A Comparative in Vitro Study. BMC Oral Health.

[4] Paratelli A., Vania S., Gómez-Polo C., Ortega R., Revilla-León M., Gómez-Polo M. (2023). Techniques to Improve the Accuracy of Complete Arch Implant Intraoral Digital Scans: A Systematic Review. J. Prosthet. Dent..

[5] Imburgia M. (2020). Continuous Scan Strategy (CSS): A Novel Technique to Improve the Accuracy of Intraoral Digital Impressions. Eur. J. Prosthodont. Restor. Dent..

[6] Iturrate M., Minguez R., Pradies G., Solaberrieta E. (2019). Obtaining Reliable Intraoral Digital Scans for an Implant-Supported Complete-Arch Prosthesis: A Dental Technique. J. Prosthet. Dent..

[7] Pozzi A., Laureti A., Tawil I., Chow J., Azevedo L., Fehmer V., Sailer I. (2025). Intra Oral Photogrammetry: Trueness Evaluation of Novel Technology for Implant Complete-Arch Digital Impression In Vitro. Clin. Implant Dent. Relat. Res..

[8] Revilla-León M., Gómez-Polo M., Drone M., Barmak A.B., Kois J.C., Alonso Pérez-Barquero J. (2025). Accuracy of Complete Arch Implant Scans Recorded by Using Intraoral and Extraoral Photogrammetry Systems. J. Prosthet. Dent..

[9] Kim Y.H., Jung B.-Y., Han S.-S., Woo C.-W. (2020). Accuracy Evaluation of 3D Printed Interim Prosthesis Fabrication Using a CBCT Scanning Based Digital Model. PLoS ONE.

[10] Gómez-Polo M., Ballesteros J., Padilla P.P., Pulido P.P., Revilla-León M., Ortega R. (2021). Merging Intraoral Scans and CBCT: A Novel Technique for Improving the Accuracy of 3D Digital Models for Implant-Supported Complete-Arch Fixed Dental Prostheses. Int. J. Comput. Dent..

[11] Peñarrocha-Diago M., Balaguer-Martí J.C., Peñarrocha-Oltra D., Balaguer-Martínez J.F., Peñarrocha-Diago M., Agustín-Panadero R. (2017). A Combined Digital and Stereophotogrammetric Technique for Rehabilitation with Immediate Loading of Complete-Arch, Implant-Supported Prostheses: A Randomized Controlled Pilot Clinical Trial. J. Prosthet. Dent..

[12] Sallorenzo A., Gómez-Polo M. (2021). Comparative Study of the Accuracy of an Implant Intraoral Scanner and That of a Conventional Intraoral Scanner for Complete-Arch Fixed Dental Prostheses. J. Prosthet. Dent..

[13] Mandelli F., Zaetta A., Cucchi A., Mangano F.G. (2020). Solid Index Impression Protocol: A Hybrid Workflow for High Accuracy and Passive Fit of Full-Arch Implant-Supported Restorations. Int. J. Comput. Dent..

[14] Mangano F.G., Bonacina M., Mandelli F., Marchiori F. (2020). Solid Index versus Intraoral Scanners in the Full-Arch Implant Impression: In Vitro Trueness Evaluation. BMC Res. Notes.

[15] Hyspler P., Strnad J., Sala R., Dostalova T. (2025). Reverse Scan Technique: A Verification Method for the Implant Position in Intraoral Scans. J. Prosthet. Dent..

[16] Hyspler P., Urbanová P., Dostalova T. (2025). Comparison of the Reverse Scan Technique with an Intraoral Scanner and the Traditional Impression Technique. J. Prosthet. Dent..

[17] Menchini-Fabris G.-B., Cosola S., Toti P., Hwan Hwang M., Crespi R., Covani U. (2023). Immediate Implant and Customized Healing Abutment for a Periodontally Compromised Socket: 1-Year Follow-Up Retrospective Evaluation. J. Clin. Med..

[18] Gallardo Y.R., Bohner L., Tortamano P., Pigozzo M.N., Laganá D.C., Sesma N. (2018). Patient Outcomes and Procedure Working Time for Digital versus Conventional Impressions: A Systematic Review. J. Prosthet. Dent..

[19] Fauroux M.-A., Germa A., Tramini P., Nabet C. (2019). Prosthetic Treatment in the Adult French Population: Prevalence and Relation with Demographic, Socioeconomic and Medical Characteristics. Rev. DÉpidémiologie Santé Publique.

[20] Stark H., Samietz S., Jordan A.R., Kuhr K., Zimmermann F., Nitschke I., Wöstmann B. (2026). Changes in Tooth Loss and Prosthetic Treatment over a 9-Year Period: Results of the 6th German Oral Health Study (DMS • 6). Quintessence Int..

